# Giant Breast Myofibroblastoma: A Rare Case of Benign Breast Tumor in a Postmenopausal Woman

**DOI:** 10.1155/cris/9930606

**Published:** 2025-08-06

**Authors:** Henry I. Lyimo, Zephania D. P. Gega, Alex B. Mashaka, Angela T. Mlole, Saida K. Abeid, Petro J. Mabindi, Nashivai E. Kivuyo, Ally H. Mwanga

**Affiliations:** ^1^Department of Surgery, Muhimbili University of Health and Allied Sciences, Dar es Salaam, Tanzania; ^2^Department of Surgery, Ocean Road Cancer Institute, Dar es Salaam, Tanzania; ^3^Department of Pathology, Muhimbili University of Health and Allied Sciences, Dar es Salaam, Tanzania; ^4^Department of Pathology, Ocean Road Cancer Institute, Dar es Salaam, Tanzania; ^5^Department of Radiology, Ocean Road Cancer Institute, Dar es Salaam, Tanzania

**Keywords:** mastectomy, menopause, myofibroblastoma

## Abstract

**Introduction:** Breast myofibroblastoma (MFB) is a relatively rare benign tumor that mimics the clinical presentation of malignant tumors of the breast. It has various morphologic variants that can be accurately diagnosed based on histopathology and immunohistochemistry staining. We report a case of MFB in a menopausal woman for pertinent clinical consideration and management.

**Case Presentation:** We report a case of a 57-year-old Tanzanian woman, who presented with a huge right breast mass for 1 year. Initial radiological findings were inconclusive, however, the tissue specimen for histology and immunohistochemical (IHC) confirmed the diagnosis. A simple mastectomy was thereafter performed as a curative therapy.

**Conclusion:** This case presentation underscores the importance of considering MFB as a potential differential for breast tumors especially in menopausal women. Tissue biopsy for histopathology and IHC staining form the cornerstone for accurate diagnosis and appropriate management.

## 1. Introduction

The clinical presentation of several benign breast conditions, common and rare, can mimic breast cancer [1–3]. Myofibroblastoma (MFB) is one of the rare benign tumors of myofibroblastic differentiation [2, 4–7]. It is described in different sites such as soft tissues, skin, axilla, lungs, lymph nodes, and breast [7, 8]. Initially, MFB was thought to have a predilection toward the male gender, however, recently these uncommon tumors have now been recognized in females with increasing frequency [9)]. There have been <90 cases of breast MFB reported to date after being first described as a distinct entity in 1987 [4, 8, 10]. It commonly presents as a small mobile and well-circumscribed mass [2, 6, 7, 11]. Cases of giant MFB greater than 10 cm in greatest dimensions have also been reported [12].

The histomorphology patterns of breast MFB show variable heterogeneity which is also evident in their clinical presentation and radiographic characteristics. This variability can lead to a wide range of differential diagnoses, particularly among other fibroepithelial and spindle cell lesions of the breast, both benign and malignant [5]. The diverse morphological variants associated with MFB contribute to the diagnostic challenges encountered in clinical practice [6, 10, 11].

An accurate diagnosis of MFB is rarely established before conducting histopathological and immunohistochemical (IHC) analyses [10]. Typically, MFB is diagnosed through core needle biopsy (CNB) and subsequently managed through local excision [2, 5]. This tumor's characteristic features include the presence of spindle cells within a collagen-rich background, low mitotic activity, and CD 34 positivity [6, 9, 10].

On IHC staining, MFB demonstrates positivity for vimentin and CD 34 variably positive for desmin and SMA, and consistently negative for S100 and CD 117 [13]. This importantly distinguishes it from other fibroepithelial and spindle cell lesions.

In this case report, we describe a rare case of a huge right breast MFB that mimics the clinical presentation of a malignant breast tumor in a postmenopausal woman. This presentation underscores the importance of histopathology and IHC in making accurate diagnosis.

## 2. Case Presentation

A 57-year-old Tanzanian woman presented with a gradual onset of a right-sided breast mass for 1 year before the hospital visit. The mass was painless and progressively increased in size. It was not associated with any nipple discharge or skin changes. There was no history of fever, night sweats, or progressive unintentional weight loss.

She attained menarche at the age of 15 years and menopause at 45 years. She gave birth to her first and only child at the age of 23 years. She reported using combined oral contraceptives (COCs) for 3 years after delivery, however, no history of using hormonal replacement therapy post menopause. Her past medical history is significant for a myomectomy performed at 37 years old, due to symptomatic uterine myoma. She denied any history of abortion, trauma, and prior surgery to the affected breast, or chest radiation. She also reported no history of alcohol consumption, tobacco smoking, or any family history of similar presentation.

On general examination, she was conscious, afebrile, with no features of anemia or icterus, and had vitals within normal limits.

Right breast examination; the right breast was enlarged compared to the left with a normal nipple–areolar complex appearance and normal overlying skin color. There was no peau d'orange or subcutaneous nodules. On palpation, there was a firm mass measuring 30 cm in its greatest dimension, occupying all four quadrants. The mass was multilobulated with well-defined margins. It was nontender and mobile, fixed to the overlying skin but not fixed to the underlying pectoralis and anterior serratus muscles or chest wall. The mass was not warm or pulsatile. There were no palpable ipsilateral axillary or supraclavicular lymph nodes.

Left breast examination was uneventful.

Systemic examination was uneventful.

The laboratory blood work-up including complete blood count (CBC), serum electrolytes, renal function tests, and liver function tests were all within normal ranges. HIV serological testing was also negative.

**Figure 1:**
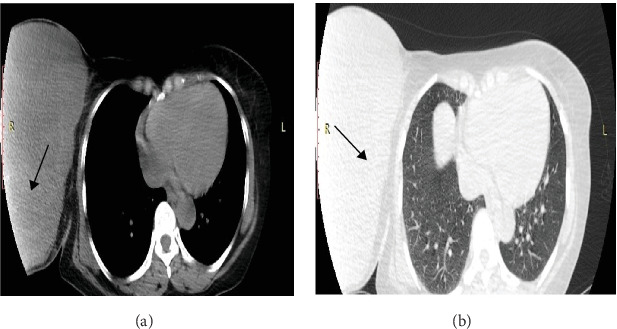
(a and b) Soft tissue and lung windows, respectively, show a heterogeneous enhanced right breast lobulated noncalcified mass measuring 32 cm (black arrows), with no suspicious ipsilateral axillary lymphadenopathy.

A contrast (IV) enhanced CT scan of the chest and abdomen; revealed a heterogeneous enhancing lobulated mass of the right breast, noncalcified, measuring 32 cms in greatest dimension with no suspicious pulmonary lesions or axillary lymphadenopathy as shown in Figure 1a,b.

**Figure 2:**
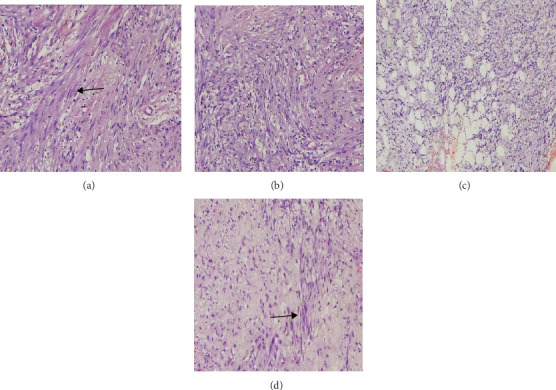
(a–d) Hematoxylin and eosin stained sections of the incisional biopsy. (a,b) Mesenchymal tumor with bland-looking spindle cell proliferation in fascicles (arrow) with interspersed eosinophilic collagen fibers, mild nuclear atypia, and low mitotic count (×40 magnification). (c,d) Spindle to stellate cells (shown by arrows) in a mucin pool (×40 magnification).

A supra-areola right breast incisional biopsy (open biopsy) was done at 2 cm from the areola complex, through an oblique incision 5 cm in length. A 3 cm portion at the maximum dimension of a firm tumor with its pseudo-capsule together with the friable parenchymal tissue was incised and taken out for histology. The estimated blood loss was 2 mls during the procedure. Histology revealed features suggestive of benign MFB as shown in Figure 2a–d and consequently, IHC tests (estrogen and progesterone receptors) were all negative.

Complete local excision of the benign mass would be the appropriate approach, however, due to the bulkiness of the tumor and difficulty in distinguishing the mass from the breast tissue; the patient underwent a right breast simple mastectomy.

Before surgery, the patient was advised and accepted for delayed breast reconstruction (TRAM Flap) since there were no frozen section services for immediate pathology results at the hospital setting where the mastectomy was performed.

**Figure 3:**
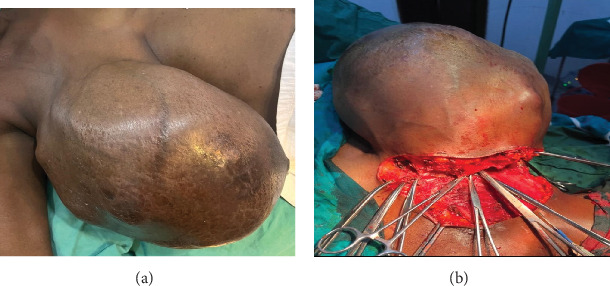
(a) A preoperative finding of a right enlarged breast, and (b) an intraoperative simple mastectomy of the right breast.

**Figure 4:**
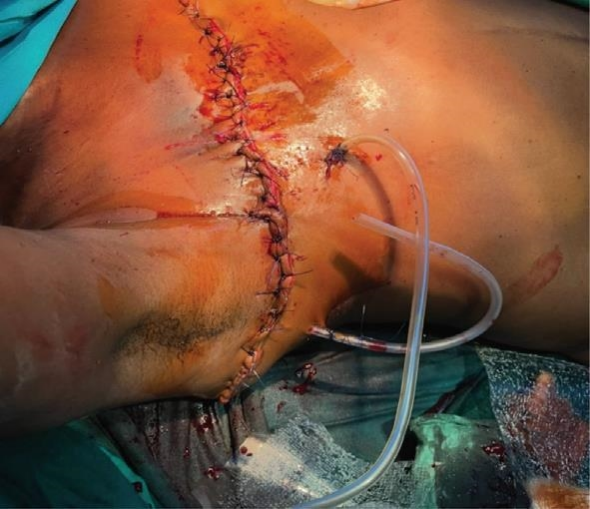
Inserted drain tubes post-simple mastectomy.

Under a sterile aseptic technique, a classic Stewart incision was made through the right breast. A huge, diffuse, vascularized, and multilobulated mass occupying all four quadrants was identified. It was mobile and adherent to the overlying skin but not the underlying muscles or chest wall. It was a whitish solid tumor with a slippery surface and abundant mucin materials. It was not distinguishable from the breast tissue with no well-demarcated surgical plane. It weighed 7 kg with a maximum dimension of 31 cm upon complete excision as shown in Figure 3a,b. A hemovac drain consisting of two perforated tubes was placed. The first tube was directed and kept underneath the superior flap and the second one was directed and kept underneath the inferior flap and both were connected outside the incisional wound with y-connector tubing to a portable hollow vacuum 350 cc drainage bottle as shown in Figure 4. The wound was closed by interrupted vertical mattress suturing technique by proline 3/0. Hypoallergenic nonwoven adhesive compressive wound dressing plastering was applied.

The drain was kept for 14 days and it was draining approximately 200 mL of serosanguinous fluid emptied per day. A total of 3 L of serous fluid had been drained upon removal of the drain tube and stitches on day 14 postoperatively.

**Figure 5:**
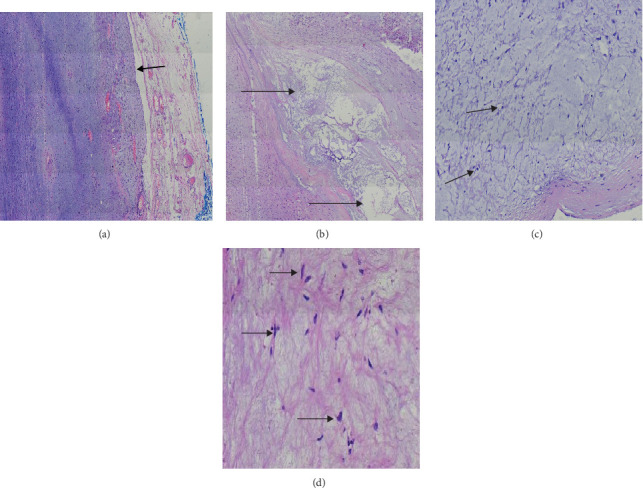
(a–d) Hematoxylin and Eosin stained sections of the tumor. (a) Section showing a well-circumscribed myxoid tumor as indicated by black arrow (×40 magnifications). (b) Section showed mucin pools (black arrows) and myxoid areas ( 40 magnifications). (c) Section showing spindle to stellate-shaped cells (black arrows) in a patternless arrangement in abundant mucin materials (×100 magnification). (d) Bland spindle to stellate cells (black arrows) in a myxoid background (×400 magnification).

**Figure 6:**
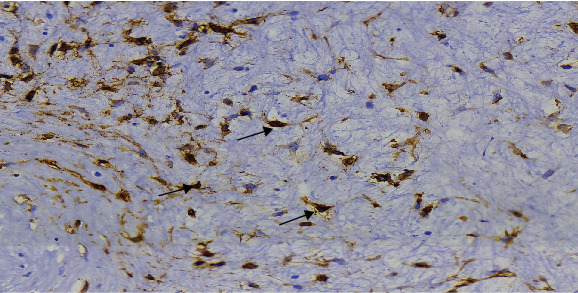
CD34 immunohistochemistry: section showing CD34 positive reactivity in tumor cells as indicated by black arrows (×400 magnification).

The histology report of the submitted right breast specimen confirmed a diagnosis of myxoid MFB as shown in Figure 5. The IHC stain for CD 34 was also positive in tumor cells as shown in Figure 6.

**Figure 7:**
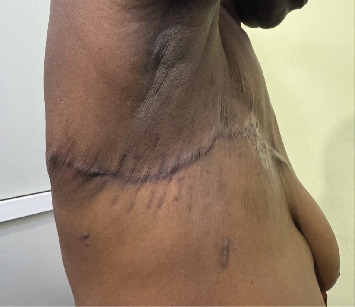
one-month post right simple mastectomy.

The patient was discharged 3 days postoperatively. After hospital discharge, the patient had daily outpatient hospital visits for post-operative wound care until the removal of drains and skin stitches on day 14. The wound site was clean and dry as shown in Figure 7. Postoperative histology results of this tumor were well explained to the patient, that the skin and all margins of resection were not involved with the tumor and there was no evidence of malignancy. She was recommended to undergo breast surgical site ultrasonography investigation to detect any sign of recurrence or abnormal regrowth every 6 months up to 2 years follow up.

## 3. Discussion

It is not uncommon for some of the breast benign tumors to have a similar clinical presentation and to some extent radiological resemblance to malignant tumors of the breast [1–3]. Breast MFB is one of the rare benign conditions that can imitate such a presentation. Despite being very rare, reported cases of breast MFB occur often in menopausal women and old men [12].

This rare condition is currently increasingly reported in females ever since the first recognized entity in 1987 [9]. Typically, it presents with a palpable mobile mass that is usually small (<3 cm) with well-circumscribed margin and encapsulated upon excision [2, 6, 7]. Often, the mass is adherent to overlying skin but not fixed to underlying muscles or chest wall [11]; rare cases of breast MFB (>10 cm) have also been reported, commonly referred to as giant breast MFB [12]. This is reflected in our case report; however, greater part of the mass was devoid of a well-circumscribed margin and encapsulation making it indistinguishable from the breast tissue.

Breast MFB is a rare benign mesenchymal lesion that shares similarities with other spindle cell tumors that exhibit overlapping histological and immunophenotypic properties, hence making its diagnosis challenging [6, 10]. As a result of this variability, differentials such as harmatoma or fibroadenoma can be confused with it [11]. Appropriate management of breast MFB requires precise and accurate diagnosis. Tissue specimen is often obtained through CNB; and thereafter sent for histopathological and IHC analyses that form the basis of distinctive and confirmatory diagnosis in this pool of histomorphologic heterogeneity [2, 5]. Bland spindle cells embedded in a collagenous background with low mitotic activity and tumor cells that show positivity toward CD 34, vimentin, SMA, desmin, negativity for S100 and CD 117 in IHC analysis form the pathognomonic features of MFB [10, 13]. This is also reflected in our case report, whereby the tumor cells had positivity towards CD 34. There is no reported malignant potential for the transformation associated with MFB; therefore, surgical excision is recommended for curative intent [11], however, in our case report due to the difficult in distinguishing the surgical plane for resection, a simple mastectomy was, therefore, done. The risk of local recurrence is also not reported especially following clear resection margins [13]. The recommended follow-up period in a patient postresection for MFB is at least 24 months [11]. Similarly, our patient was recommended to undergo breast surgical site 6 monthly USG to detect any early signs of recurrence during the 2 year follow-up.

## 4. Conclusion

Breast MFB remains one of the rare benign breast tumors that mimic the clinical presentation of breast malignant tumors. Prompt CNB for histopathology confirms its benign nature along with immunohistochemistry allowing its distinction among many other mesenchymal variants that have similar presentation and clinical course. A complete surgical excision forms the basis of curative therapy in these tumors.

## Data Availability

The authors have nothing to report.
